# Extensive Variation in the Activities of *Pseudocerastes* and *Eristicophis* Viper Venoms Suggests Divergent Envenoming Strategies Are Used for Prey Capture

**DOI:** 10.3390/toxins13020112

**Published:** 2021-02-02

**Authors:** Bianca op den Brouw, Francisco C. P. Coimbra, Lachlan A. Bourke, Tam Minh Huynh, Danielle H. W. Vlecken, Parviz Ghezellou, Jeroen C. Visser, James S. Dobson, Manuel A. Fernandez-Rojo, Maria P. Ikonomopoulou, Nicholas R. Casewell, Syed A. Ali, Behzad Fathinia, Wayne C. Hodgson, Bryan G. Fry

**Affiliations:** 1 Venom Evolution Lab, School of Biological Sciences, University of Queensland, St Lucia, QLD 4072, Australia; francisco.cp.coimbra@gmail.com (F.C.P.C.); l.bourke@uq.net.au (L.A.B.); jeroencvisser@hotmail.com (J.C.V.); j.dobson@uq.edu.au (J.S.D.); 2 Monash Venom Group, Faculty of Medicine, Nursing & Health Sciences, Monash University, Clayton, VIC 3800, Australia; tlhuy3@student.monash.edu (T.M.H.); wayne.hodgson@monash.edu (W.C.H.); 3 Department of Animal Science and Health, Institute of Biology Leiden, 2333 BE Leiden, The Netherlands; daniellevlecken@gmail.com; 4 Medicinal Plants and Drugs Research Institute, Shahid Beheshti University, 1983969411 Tehran, Iran; p.ghezellou@gmail.com; 5 Institute of Inorganic and Analytical Chemistry, Justus Liebig University Giessen, 35392 Giessen, Germany; 6 Madrid Institute for Advanced Studies in Food, E28049 Madrid, Spain; manuel.fernandez@imdea.org (M.A.F.-R.); maria.ikonomopoulou@imdea.org (M.P.I.); 7 Institute for Molecular Bioscience, The University of Queensland, Brisbane, QLD 4072, Australia; 8 Centre for Snakebite Research & Interventions, Liverpool School of Tropical Medicine, Liverpool L3 5QA, UK; Nicholas.Casewell@lstmed.ac.uk; 9 HEJ Research Institute of Chemistry, International Centre for Chemical and Biological Sciences (ICCBS), University of Karachi, Karachi 75270, Pakistan; dr.syedabidali@gmail.com; 10 Department of Biology, Faculty of Science, Yasouj University, 75914 Yasouj, Iran; bfathinia@gmail.com

**Keywords:** *Pseudocerastes*, *Eristicophis*, venom, haemotoxic, neurotoxic, cytotoxic, venom variation

## Abstract

Snakes of the genera *Pseudocerastes* and *Eristicophis* (Viperidae: Viperinae) are known as the desert vipers due to their association with the arid environments of the Middle East. These species have received limited research attention and little is known about their venom or ecology. In this study, a comprehensive analysis of desert viper venoms was conducted by visualising the venom proteomes via gel electrophoresis and assessing the crude venoms for their cytotoxic, haemotoxic, and neurotoxic properties. Plasmas sourced from human, toad, and chicken were used as models to assess possible prey-linked venom activity. The venoms demonstrated substantial divergence in composition and bioactivity across all experiments. *Pseudocerastes urarachnoides* venom activated human coagulation factors X and prothrombin and demonstrated potent procoagulant activity in human, toad, and chicken plasmas, in stark contrast to the potent neurotoxic venom of *P. fieldi*. The venom of *E. macmahonii* also induced coagulation, though this did not appear to be via the activation of factor X or prothrombin. The coagulant properties of *P. fieldi* and *P. persicus* venoms varied among plasmas, demonstrating strong anticoagulant activity in the amphibian and human plasmas but no significant effect in that of bird. This is conjectured to reflect prey-specific toxin activity, though further ecological studies are required to confirm any dietary associations. This study reinforces the notion that phylogenetic relatedness of snakes cannot readily predict venom protein composition or function. The significant venom variation between these species raises serious concerns regarding antivenom paraspecificity. Future assessment of antivenom is crucial.

## 1. Introduction

Snakebite envenoming causes the death or debilitation of hundreds of thousands of people annually [1]. To address this global health crisis, a better understanding of the evolution, biology, ecology, venom, and envenoming statistics of medically important snakes is crucial. Despite this, numerous knowledge gaps surrounding many venomous species still exist. These gaps are particularly associated with developing regions such as the Middle East, which has been subject to extended periods of political instability and ongoing tension. The data-deficient nature of the Middle Eastern herpeto-faunal literature is exemplified by the relatively recent description of a third species of viperid snake from the genus *Pseudocerastes* (Boulenger, 1896), the spider-tailed viper *Pseudocerastes urarachnoides* (Viperidae: Viperinae) (Bostanchi, Anderson, Kami, and Papenfuss, 2006) from western Iran [2,3].

The genus *Pseudocerastes*, comprised of *P. fieldi* (Schmidt, 1930), *P. persicus* (Duméril, Bibron and Duméril, 1854), and *P. urarachnoides* is sister to the monotypic species *Eristicophis macmahonii* (Alcock and Finn, 1897), which together form a clade colloquially named the “desert vipers.” The clade is proposed to have split from the most recent common ancestor of the remaining Eurasian viper genera (*Vipera*, *Daboia*, *Macrovipera*, and *Montivipera*), to which they are sister, around 25–33 million years ago (Mya) [4,5,6]. Further speciation events within the clade occurred approximately 16–18 Mya (*E. macmahonii*), 12 Mya (*P. fieldi*), and 8 Mya (*P. persicus* and *P. urarachnoides*) [4,7]. Distribution data on these species is incomplete, though they are mostly found throughout the Middle East: *E. macmahonii* in the Baluchistan (covering the Iran-Afghanistan-Pakistan border) and the Thar (northwest India) deserts [8]; *P. urarachnoides* in western Iran and eastern Iraq [9,10,11]; *P. persicus* from northeast Iraq to western Pakistan (including southern Afghanistan and Iran), as well as some isolated populations in Oman and the United Arab Emirates (UAE) [12,13]; and *P. fieldi* from northern Saudi Arabia, through the south of Israel, Jordan, and Syria, and across Iraq and southern Iran [3,14]. There appears to be limited range overlap between these species, though sympatry exists between *P. fieldi* and *P. urarachnoides* and between *P. urarachnoides* and *P. persicus* in the northern and southern edges of *P. urarachnoides*’ range in western Iran, respectively [13,15]. It is thus posited that all three species of *Pseudocerastes* likely exist in sympatry within western Iran [15].

**Figure 1:**
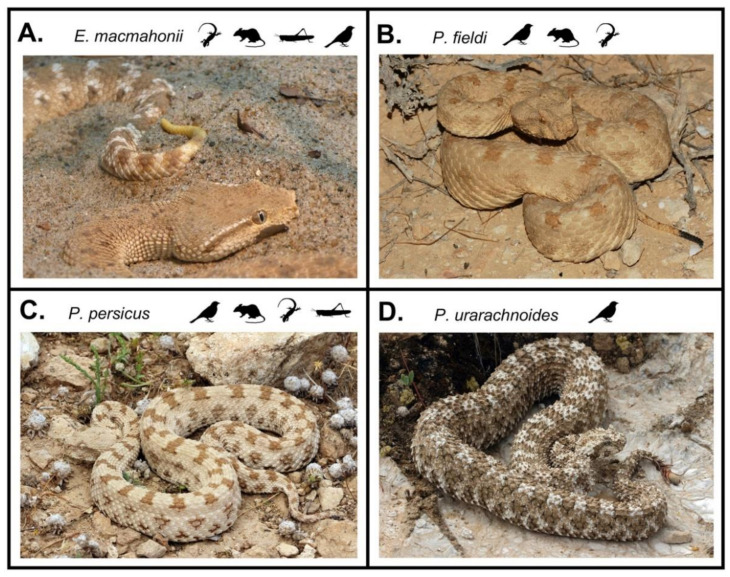
Image and corresponding diet (prey animals depicted in snakes’ order of preference) of (**A**) *Eristicophis macmahonii* (image: Tim Vickers); (**B**) *Pseudocerastes fieldi* (image: Gabriel Martinez Del Marmol); (**C**) *Pseudocerastes persicus* (image: Laura and Bobby Bok); (**D**) *Pseudocerastes urarachnoides* (image: Laura and Bobby Bok).

Field studies on the ecology of these snakes are scarce, and information on diet is largely restricted to that of adult snakes. From what is known, all species inhabit arid environments. *Eristicophis macmahonii* can be found in sandy deserts with shifting dunes and limited vegetation [16], and conceals its body within the loose sand to ambush such prey items as lizards, rodents, arthropods, and occasionally birds (Figure 1A) [2,8,17,18]. *Pseudocerastes fieldi* occupies a diversity of arid environments, though their habitat preferences appear to differ between populations across the range of this species. For example, Israeli populations tend to be associated with shrubs in semi-deserts with harder substrates and level ground [19], whereas those found in Jordan are most common in the black basalt deserts with large rocks amongst loose sandy soils and little to no vegetation [20]. Prey items include birds, mammals, and lizards, with a possible preference for birds on account of feathers being abundant in their faeces (Figure 1B) [8,19]. However, of note is the readiness in which captive specimens reportedly consume carcasses, at times even in favour of live animals. Dead migratory birds may thus form an important proportion of the diet of some populations of *P. fieldi* [19]. *Pseudocerastes persicus* occupies a variety of habitats, including semi-deserts with varied substrates including sand and rock, as well as arid plains with vegetation, steep rocky terrain, and rugged slopes typically over 500 m above sea level (asl) [2,12,21]. For example, in the Hajar Mountains in Oman, this species occurs in rocky environments between 500 and 2500 m asl, which are subject to relatively cold and humid conditions [22]; however, in the United Arab Emirates it has also been found as low as 195 m asl [23]. A generalist feeder, *P. persicus* predates upon birds, mammals, lizards, and occasionally arthropods (Figure 1C) [2,8]. *Pseudocerastes urarachnoides* can be found on the sheer faces of rocky hills with sparse vegetation [9,24], and adults use a highly derived spider-like tail adaptation to lure birds on which they appear to feed exclusively (Figure 1D) [9,10,24,25]. The caudal lure is not developed in juveniles but forms progressively as the snakes grow. All desert vipers are assumed to employ caudal luring on the basis of featuring coloured or modified tail tips; however, this behaviour has only been documented in *P. urarachnoides* [9,15]. The tail of *P. urarachnoides* is the most specialised and elaborate of the clade—of any reptile, even—perhaps as a reflection of its narrow ecological niche.

Despite their relatively close geographical proximity and apparently similar diets—factors that are commonly posited to influence the extent of venom variation within a clade—the venoms of these species differ considerably. For example, *E. macmahonii* venom possesses procoagulant properties [26] while *P. persicus* is anticoagulant [27] and the phospholipase-dominated venom of *P. fieldi* is neurotoxic [27]. In a prior proteomic study of the venoms, the authors posited that *P. persicus* represents an intermediate condition between the apotypic venom of *P. fieldi* and the plesiotypic venom of *E. macmahonii* [21]. However, a comparative proteomic or functional analysis of all four desert vipers’ venoms has not been conducted, and virtually nothing is known about the venom of *P. urarachnoides*.

To address this knowledge gap, this study analysed the venoms of the desert viper clade via proteomics and functional venomics. Venom protein profiles were visualised via one-dimensional gel electrophoresis (1D SDS-PAGE) and venom toxins of *P. urarachnoides* were identified by two-dimensional gel electrophoresis (2D SDS-PAGE) followed by liquid chromatography/mass spectrometry (LC/MS-MS). Venom-induced cytotoxicity was assessed by incubating the venoms with human melanoma (MM96L), neonatal foreskin (NFF), and pancreatic tumour (PaTu-T) cell lines for 24 h and visualising cell death via light microscopy. Neurotoxic venom properties were investigated by chick biventer cervicis assays. The haemotoxic activities of the venoms were evaluated using an automated coagulation analyser, by thromboelastography, and by measuring venom-induced FX and prothrombin cleavage. Prey-linked venom activity was investigated via thromboelastography using plasma sourced from three model animal groups: toad (amphibian), chicken (avian), and human (mammalian). A broad, exploratory overview of findings is discussed in the context of pathophysiology and implications for snakebite envenoming, conjectured ecological significance, and directions for future research.

## 2. Results and Discussion

### 2.1. Venom Proteomics

**Figure 2:**
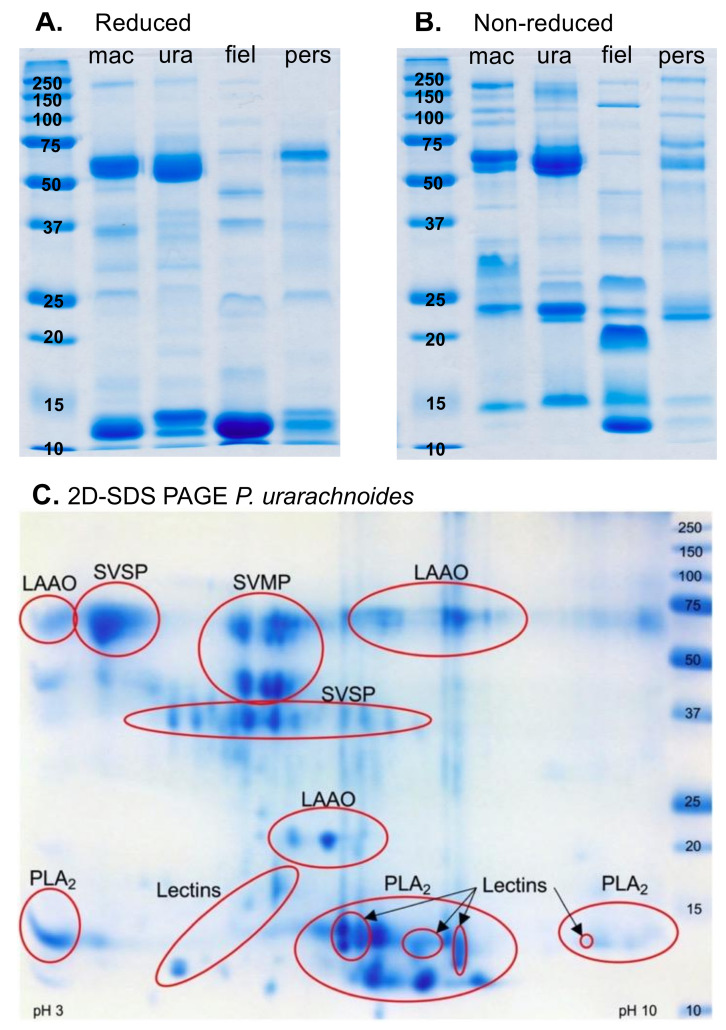
Sodium dodecyl sulphate polyacrylamide gel electrophoresis (SDS-PAGE) of (**A**) 20 µg *Pseudocerastes* and *Eristicophis* venoms run under reducing and denaturing conditions; (**B**) 20 µg *Pseudocerastes* and *Eristicophis* venoms run under denaturing conditions; and (**C**) 200 µg *Pseudocerastes urarachnoides* venom run under reducing, denaturing, and isoelectric focusing (pI) conditions. Mac = *E. macmahonii*; ura = *P. urarachnoides*; fiel = *P. fieldi*; pers = *P. persicus;* SVSP = Snake venom serine protease; LAAO = L-amino acid oxidase; PLA_2_ = Phospholipase A_2_; SVMP = Snake venom metalloprotease.

The 1D SDS-PAGE comparisons revealed substantial differences in the venom protein composition of the species under study (Figure 2). Each species exhibited a diversity of venom components ranging from ~12 kDa to >150 kDa in size, and unique bands were observed in each of the venoms. These differences were particularly evident in the non-reduced gel profiles (Figure 2B). While banding patterns differ, these gels revealed a similar (though differential) complexity between *P. fieldi* and *P. persicus* venoms, despite previous SDS-PAGE analyses reporting “very few” protein bands in the former venom compared with “close to 30” in the latter [27]. It must be noted, however, that <20 kDa components (predominantly phospholipases (PLA_2_s) [21]) clearly dominate *P fieldi* venom, and additional banding is generally weak throughout both venom profiles. In contrast, the protein composition of *P. urarachnoides* and *E. macmahonii* venoms possessed a number of intense protein bands that were concentrated in both the upper (>50 kDa) and the lower (<25 kDa) mass regions of the 1D gels.

A mass spectrometry analysis of 2D gel spots for *P. urarachnoides* revealed the expression of numerous snake venom metalloproteases (SVMP), snake venom serine proteases (SVSP), and L-amino acid oxidases (LAAO) isoforms in the mid (~20–45 kDa) and high (>50 kDa) molecular weight regions, and an array of PLA_2_ and lectin isoforms in the region below 20 kDa (Figure 2C). These toxin classes are abundant in the venoms of most viperids, including *E. macmahonii*. However, SVSPs and LAAOs are reduced or absent from the venoms of both *P. persicus* and *P. fieldi*, and SVMPs greatly reduced in the latter [21]. The venom composition of *P. urarachnoides* therefore appears to share greater similarities with that of *E. macmahonii* (most recent common ancestor (MRCA) 16.5 Mya) than that of its sister species, *P. persicus* (MRCA 8 Mya) [4]. Notably, however, the PLA_2_ profile of *P. urarachnoides* venom was dominated by neutral phospholipase isoforms, which are absent or greatly reduced from *E. macmahonii* and *P. persicus* venoms, respectively, though similarly abundant in that of *P. fieldi* [21].

Of additional interest is the differential banding complexity between reduced and non-reduced profiles for all species. This suggests that protein complexes and dimers or trimers are abundant in these venoms. A heterodimeric PLA_2_ complex, consisting of one basic and one acidic phospholipase subunit, has been described as the main toxic component of *P. fieldi* venom [28,29]. The comparative protein profiles of the reduced and non-reduced gels in the lower regions (<25 kDa) corroborate the presence of this complex in *P. fieldi* and also alludes to the presence of such complexes in each venom (see *Neurotoxicity* section for further discussion).

### 2.2. Venom-Induced Cytotoxicity

**Figure 3:**
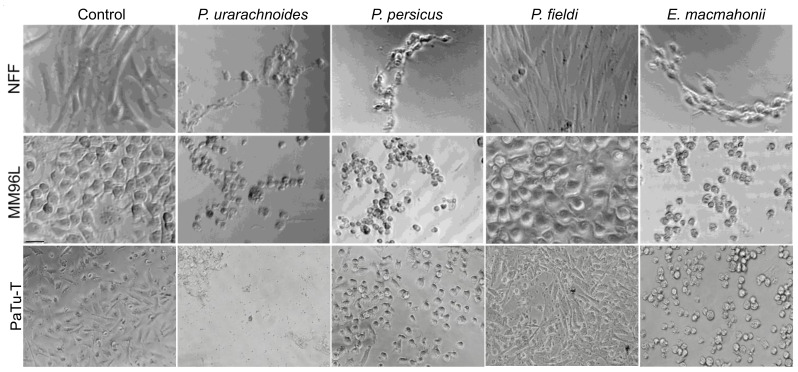
Cytotoxic effects of 10 μg/mL *Pseudocerastes* and *Eristicophis* venoms on human pancreatic cancer (PaTu-T), melanoma (MM96L), and skin (NFF) cells after 24 h of incubation, visualised via light microscopy at 10× magnification. Rounding and detachment of cells from the well plate (comparative to the control) indicates onset and progression of cell sickness and death.

The cytotoxic properties of 10 μg/mL of the venoms in NFF (5000 cells/well), MM96L (3000 cells/well), and PaTu-T (at 80% confluence) cells were visually assessed by light microscope imaging at 10× magnification after 24 h of incubation. Though activities of the venoms differed between cell lines, *P. urarachnoides*, *P. persicus*, and *E. macmahonii* venoms all variably impacted cell health (Figure 3). However, the venom of *P. urarachnoides* appeared to show the most consistent and detrimental effect across all cell lines. It is thus posited to possess the strongest cytotoxic effects of all the venoms, followed by *E. macmahonii*, though future quantification of the venoms is needed for confirmation. Both venoms contain large quantities of serine proteases and metalloproteases along with smaller quantities of L-amino acid oxidases [21], each of which are commonly associated with cellular and tissue degradation. When all cell lines are viewed in unison, *P. persicus* venom demonstrated moderate to strong cytotoxic properties, while *P. fieldi* venom cytotoxicity was residual. L-amino acid oxidases are absent from these latter two venoms, and expression of serine proteases and metalloproteases is greatly reduced compared to the venoms of *P. urarachnoides* and *E. macmahonii* [21]. The variable cytotoxic activities observed in the microscopy assessment may therefore reflect this differential expression of commonly cytotoxic toxin groups.

### 2.3. Venom-Induced Neurotoxicity

**Figure 4:**
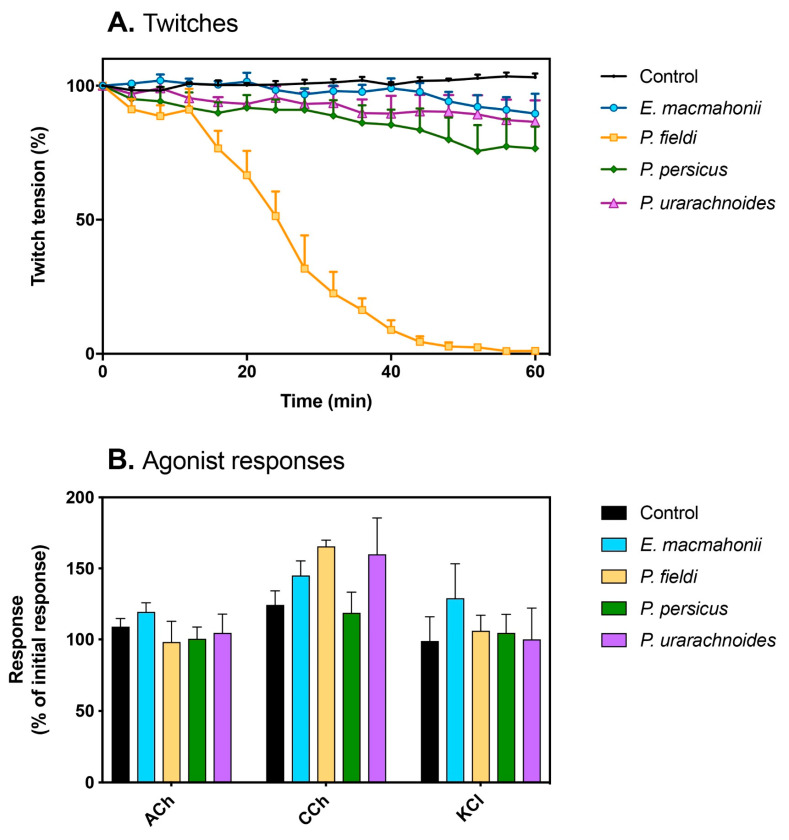
(**A**) The effects of 10 µg/mL *Pseudocerastes* and *Eristicophis* venoms on indirect twitches of the chick biventer muscle; (**B**) Change in contractile response following *post hoc* addition of agonists acetylcholine (ACh; 1 mM), carbachol (CCh; 20 µM), and potassium chloride (KCl; 40 mM).

The venoms were tested for neurotoxicity at the neuromuscular junction by measuring their ability to abrogate chick biventer cervicis contractions at a concentration of 10 µg/mL. Of all the venoms, only *P. fieldi* abolished twitches, with full inhibition occurring after approximately 50 min (Figure 4A). Following the addition of acetylcholine (ACh), carbachol (CCh), or potassium chloride (KCl), there was no reduction in agonist responses in the presence of *P. fieldi* venom (Figure 4B), which indicates presynaptic neurotoxicity. A neurotoxic PLA_2_ complex from *P. fieldi* venom has been well-characterised and acts at the presynaptic site to induce irreversible neuromuscular paralysis [30]. While the basic subunit appears to be that which confers lethality, its potency is greatly enhanced in the presence of the weakly toxic acidic subunit. The acidic subunit is thus conjectured to potentiate the lethal activity of the basic phospholipase by acting as a chaperone, inhibiting non-specific binding and increasing its affinity for its molecular target [28,31]. The diffusion of such neurotoxic phospholipases throughout a biological system may be hindered by venom components that disrupt haemostatic balance, such as SVMPs, by inducing local cell adhesion, inflammation, affecting blood flow rate, and so on. Such interplays may be an explanation for the inverse trend between SVMPs and PLA_2_s that can be commonly observed in viper venoms, including *P. fieldi*.

There was a significant difference in the neurotoxic activity between the venoms (one-way ANOVA: F_4,15_ = 174.7, *p* < 0.0001). Despite this, at the 60 min time point, each of the other three venoms reduced twitch height by a small but significant degree compared to the negative control (Tukey’s post hoc analysis; mean reduction by *E. macmahonii*: 13.5%, *p* = 0.0469; *P. urarachnoides*: 16.6%, *p* = 0.0199; *P. persicus*: 26.5%, *p* = 0.0002). As this reduction was minor, it could be explained by either of two scenarios. Other toxin components may be inducing tissue damage or fatigue, which over time result in a reduction of twitch height; alternatively, low levels of neurotoxins may be expressed in these venoms. While neither can be unequivocally asserted in the absence of comparative transcriptomics, modest evidence in this study suggests that the latter may be the case. The cytotoxicity observations indicate that *P. urarachnoides* and *E. macmahonii* possess the greatest cytopathic activities, while those of *P. persicus* and *P. fieldi* were moderate to low, respectively. Should tissue damage be occurring by cell death, a similar relationship in reduction of neurotoxicity would be expected. However, in the neurotoxicity assays, the inverse is observed, whereby the venoms with strongest cytotoxicity induced the smallest reduction in twitch height. Furthermore, cytotoxic activities are notoriously slow-acting. Cell death in all three cell lines was observed after 24 h of incubation compared to the 1-h chick biventer assay. Additionally, should cytotoxic damage have occurred to the tissue, this would likely be reflected in an increase in base-line tension and a reduction of response following the post hoc addition of KCl. These factors suggest that the impact of the cytotoxins on the chick biventer cervicis are limited. Finally, each venom possesses an array of PLA_2_ isoforms, some complexes of which are documented as possessing neurotoxic properties [28,31]. Cumulatively, this alludes to the strong possibility that these modest twitch reductions reflect the activity of a low concentration of neurotoxins. Should this be the case, neurotoxic PLA_2_s may have been present in the last common ancestor of this clade.

While the neurotoxic content of *P. persicus* venom appears low compared to *P. fieldi*, very weak venom concentrations are tested in these assays. For example, 10 µg/mL is probably up to 200 times weaker than the venom concentration that may result in the body of a 200-g prey animal following envenoming [32]. It is difficult to speculate upon the susceptibility of different animals to venoms, though it could be reasonably argued that a weak neurotoxic effect at 10 µg/mL in an in vitro model may offer a significant contribution to prey subjugation at biologically realistic concentrations. Neurotoxic activity has been conjectured to offer an advantage in subduing prey with high escape potential, which could reduce the chance of prey loss following envenoming by limiting distance travelled [33]. However, this may be contingent upon a bite-and-release strategy, which is variably employed by *P. fieldi* and *P. persicus* depending on prey species and is not employed by *P. urarachnoides* or *E. macmahonii* [8].

### 2.4. Venom-Induced Coagulant Activity

#### 2.4.1. Coagulation Time, Strength, and Potency

**Figure 5:**
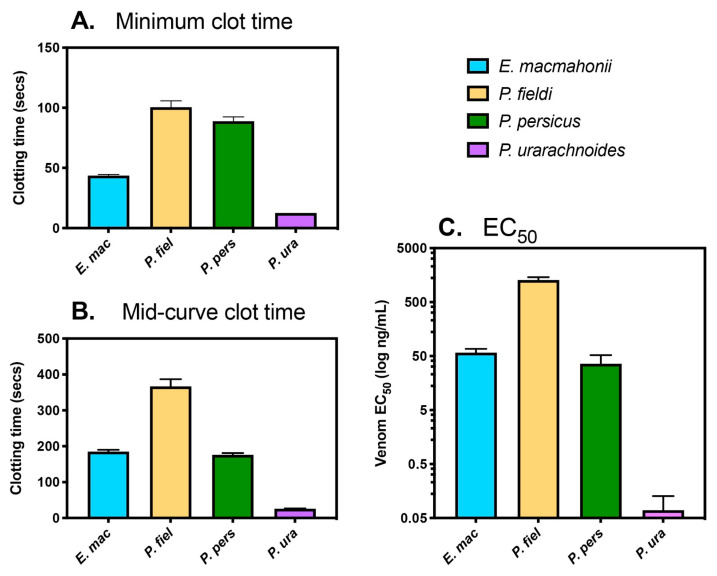
Coagulant activity of *Pseudocerastes* and *Eristicophis* venoms on human plasma in relation to (**A**) Maximal activity (minimum clot time); (**B**) Strength of activity (mid-curve clot time); and **(C)** Relative venom potency (EC_50_). Mean ± SD, *n* = 3.

The influence of the venoms on the coagulation of human plasma was tested by incubating plasma with increasing concentrations of venom until the horizontal asymptote (minimum clot time) was reached. The minimum clot time (Figure 5A) represents the strength of the venoms’ clotting activity—i.e., their maximal clot-inducing potential. As a rule-of-thumb, if a venom takes longer than 80 s to induce clotting at its horizontal asymptote (i.e., its maximal activity), the resultant clots are likely to be poorly formed and friable and the venom is not considered to be procoagulant. The mid-curve clot time (Figure 5B) is the curve point between minimal and maximal activity within these concentration curves. This value is a proxy for the venom toxins’ affinities for their plasma targets, represented by the proportional change in clot time per unit of venom over the curves’ concentration range (between 0.05–30 µg/mL). A smaller relative shift in clot time with decreasing venom concentrations suggests greater toxin affinities. The EC_50_ (50% of the Effective Concentration) (Figure 5C) is the concentration of venom that induces a clotting response at the mid-point between no effect (i.e., the blank control clotting time of 584 ± 41.3 s) and maximal effect (minimum clot time), representing venom potency. When viewed in parallel, these parameters provide greater resolution of differences in coagulant activity between venoms. In particular, the interplay between strength of activity (clotting times) and potency are an important consideration regarding envenoming.

All venoms required the presence of calcium for their activity. Minimum clotting time was achieved by 20 µg/mL of venom for *P. urarachnoides* (13 ± 0.1 s) and 30 μg/mL for *E. macmahonii* (47 ± 0.1 s), *P. fieldi* (101 ± 5.3 s), and *P. persicus* (89 ± 3.7 s) (Figure 5A). These clotting times varied significantly across the clade (one-way ANOVA: F_3,7_ = 543, *p* < 0.0001). While neither *P. fieldi* nor *P. persicus* were procoagulant under the aforementioned rule-of-thumb, plasma incubated with these venoms clotted at a significantly faster rate than the plasma blank control (Tukey’s: *p* < 0.0001 for each venom). This weak clotting activity suggests that they contain procoagulant toxins in their venom, albeit at very low levels. The coagulant activity of *P. fieldi* venom was particularly weak (EC_50_ = 1288.00 ng/mL, 99% CI [1144.00, 1450.00] (Figure 5B); mid-curve clotting time = 367 ± 20.0 s (Figure 5C)), though this remains consistent with case reports documenting instances of mild coagulopathy [8].

In contrast, the procoagulant activity of *P. urarachnoides* venom was very strong, with clotting times comparable to those achieved by Sri Lankan Russell’s viper venom (*Daboia russelii*) (13.5 s)—a species infamous for its rapid procoagulant activity (see Supplementary Data). The extremely potent venom of *P. urarachnoides* (EC_50_ = 0.07 ng/mL, 99% CI [0.04, 0.13]) also retained its procoagulant activity with decreasing venom concentrations. At its mid-curve clotting point, 0.4 ± 0.0 µg/mL venom induced a clot in 26 ± 0.5 s—a doubling in clot time despite a 50-fold decrease in venom concentration. While there is virtually no data available regarding snakebite envenoming by *P. urarachnoides*, these findings indicate that a bite by this species is likely to result in clinically relevant coagulopathy.

While *E. macmahonii* venom possessed procoagulant activity, it was significantly weaker than that of *P. urarachnoides* (Tukey’s: *p* < 0.0001). Furthermore, the venom’s procoagulant toxins appeared to have a relatively low affinity for their plasma targets as clotting times declined quickly with decreasing venom concentrations (mid-curve clot time = 185 ± 5 s by 0.4 ± 0.0 µg/mL venom; a 4-fold reduction in speed). For example, despite that *E. macmahonii* venom exhibited a significantly faster maximal clotting speed compared to that of *P. persicus* (unpaired t-test: t (4) = 20.79, *p* < 0.00001), there was no significant difference between their mid-curve clot times (*P. persicus* mid-curve clot time = 176 ± 5 s by 0.4 ± 0.1 µg/mL venom; unpaired t-test: t (4) = 1.273, *p* = 0.2720). Accordingly, the potency of *E. macmahonii* venom (EC_50_ = 58.25 ng/mL, 99% CI [48.66, 68.19]) was lower than that of *P. persicus* (EC_50_ = 36.16 ng/mL, 99% CI [21.39, 52.19]), though this difference was not significant (unpaired t-test: t (4) = 2.069, *p* = 0.1074). Overall, the procoagulant activity of *E. macmahonii* venom could best be described as moderate. These results also suggest that the posited low levels of procoagulant toxins within *P. persicus* venom may have a high affinity for plasma components. Such coagulant activity could therefore be an important consideration surrounding *P. persicus* envenoming as it may contribute to coagulopathy, even at low venom doses.

#### 2.4.2. Activation of Factor X and Prothrombin

**Figure 6:**
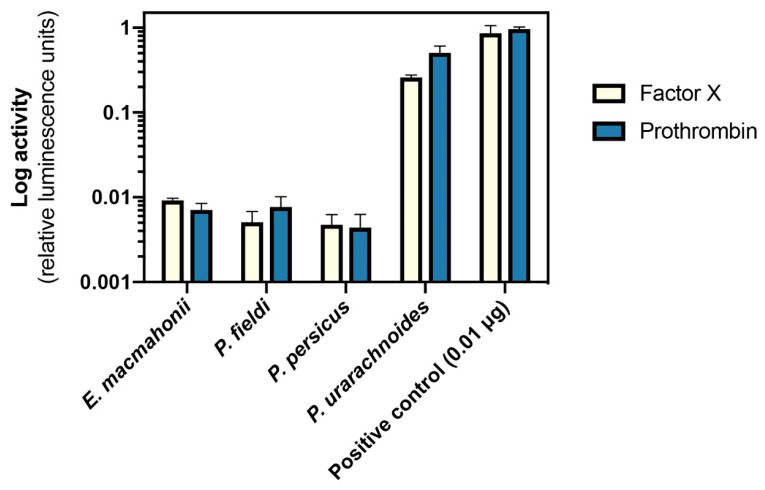
Activity of 0.01 µg human blood factors X and prothrombin by 0.01 µg of *Pseudocerastes* and *Eristicophis* venoms, measured via fluorometry. Plots show log activity, normalised to positive controls; mean ± SD, *n* = 5. Activation is calculated as the relative luminescence units (RLU) emitted by wells containing venom + zymogen + substrate after subtraction of venom + substrate control RLU. Positive controls = 0.01 µg of human Factor Xa and thrombin.

The ability of the venoms to cleave the zymogens FX and prothrombin was tested by incubating 0.01 µg of venom with 0.01 µg of human FX and human prothrombin and measuring cleavage activity via fluorometry. *Pseudocerastes urarachnoides* venom readily cleaved both prothrombin and FX (Figure 6), therefore likely representing the predominant factors through which this species induces coagulation. *Pseudocerastes persicus* and *P. fieldi* demonstrated an extremely low degree of zymogen activation, which aligns with the very weak procoagulant activity of these venoms. *Eristicophis macmahonii*, however, possessed FX and prothrombin activation at a similarly low level, discordant with its significantly stronger clotting times. This suggests that an alternative procoagulant mechanism may by stimulated by this venom, such as FV activation, and further indicates substantial divergence in toxin activity between *E. macmahonii* and *P. urarachnoides* despite superficial similarities in their protein profiles and procoagulant activity. It would be informative to test a broader array of venom-zymogen interactions in the future in order to elucidate the differences in procoagulant toxin activities of these venoms.

#### 2.4.3. Thromboelastography

The clot kinetics of human, toad, and chicken plasmas when incubated with venom were tested via thromboelastography (TEG). Baseline parameters were established by measuring the spontaneous clotting of plasmas without venom. To assess influence of the venoms on clot dynamics, data were normalised to these controls (spontaneous control = 0%) and potential maxima (100%). Three parameters are presented: “split point,” the time interval of clot initiation (minutes); “angle,” the rate of clot formation (degrees); and “maximum amplitude,” the elasticity (and thus strength) of the resultant clot (millimetres). Statistical analyses compared venom-induced clotting values with the spontaneous controls, unless otherwise stated.

**Figure 7:**
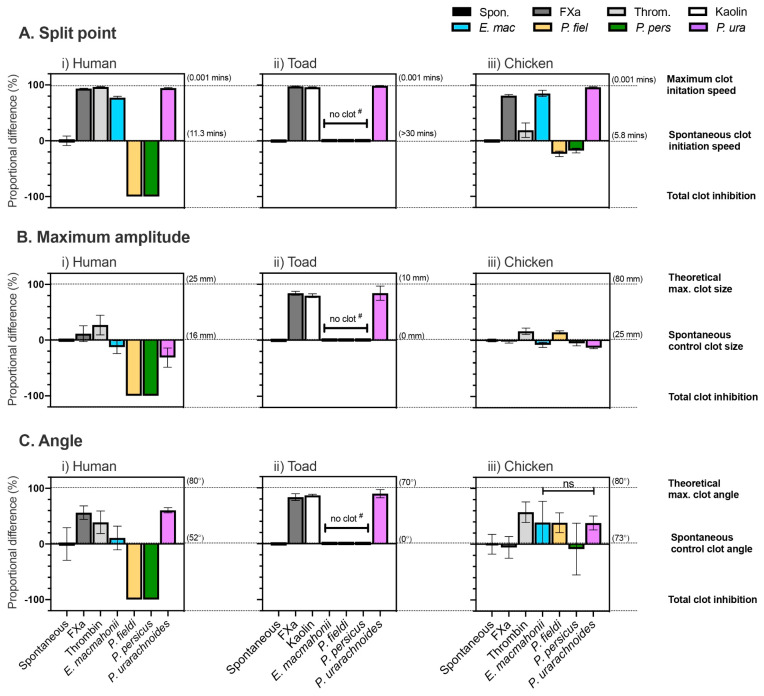
Proportional difference (%) of the clotting parameters (**A**) Split point, (**B**) Maximum amplitude, (**C**) Angle) of (i) human, (ii) toad, and (iii) chicken plasmas when incubated with or without 10 μg/mL *Pseudocerastes* and *Eristicophis* venoms. Data (mean ± SD, *n* = 4) were normalised to spontaneous clotting controls (0%) and theoretical maxima (100%). ^#^ Spontaneous clotting did not occur in toad plasma. Therefore, *post hoc* analyses (Figure 8) were run with these venoms to determine whether the absence of clotting was an anticoagulant effect or no effect.

Factor X is highly conserved between the taxa used as models, and this was reflected by human FXa activating clotting in each plasma sample (Figure 7).

**Figure 8:**
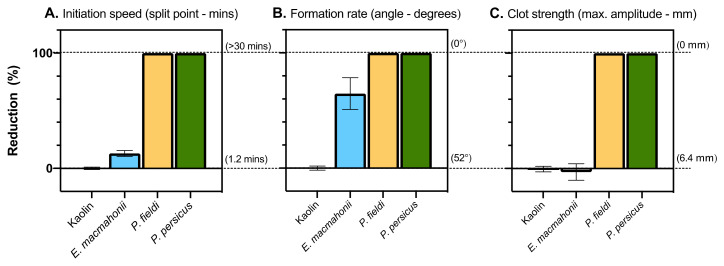
Reduction (%) of: (**A**) initiation speed, (**B**) formation rate, and (**C**) clot strength of toad plasma by *Pseudocerastes* and *Eristicophis* venoms (10 µg/mL, 30-min incubation) following the addition of clotting activator kaolin. Data (mean ± SD, *n* = 3) normalised to clotting values obtained by kaolin-only control (0%).

Human thrombin, however, was unable to clot toad plasma (see Supplementary Data), which indicates that the cleavage sites of toad thrombin, FV, and/or fibrinogen must differ sufficiently from those of human. Kaolin, which induces clotting via its negative charge, was instead used as a control for toad plasma. An additional difference within the toad plasma was evident in its lack of spontaneous clotting. Therefore, if a venom did not induce clotting in the toad plasma, it was unclear whether there was an anticoagulant effect or simply no effect. To circumvent this issue, post hoc testing was conducted on venoms that did not induce clotting by incubating toad plasma and venom, adding kaolin after 30 min, and then measuring the ensuing clot for an additional 30 min in order to determine the extent of clotting inhibition by the venoms (Figure 8).

The venom of *E. macmahonii* did not induce coagulation in toad plasma. Post hoc testing indicated that the venom had a weak to moderate anticoagulant effect, slowing down clot initiation speed by only 13 ± 2.6% and slowing clot formation rate by 65 ± 13.7%. Clot strength was unaffected (unpaired t-test: t (4), *p* = 0.5823). The venom did, however, demonstrate rapid procoagulant activity in both chicken and human plasmas (85 ± 5.7% and 77 ± 2.5% increase in clot initiation speed, respectively). The venom’s effect on chicken plasma was slightly greater than on human (Unpaired t-test: t (6) = 2.471, *p* = 0.0484). While the venom elevated the rate of clot formation in both plasmas, these increases were not significant (Chicken: 54 ± 29%; unpaired t-test: t (6) = 1.850, *p* = 0.1138. Human: 11 ± 21.3%; unpaired t-test: t (6) = 0.5963, *p* = 0.5728). The venom’s effects on clot strength were minimal (Chicken: −8 ± 4.7%; unpaired t-test: t (6) = 2.954, *p* = 0.0255. Human: −13 ± 11.6%; unpaired t-test: t (4) = 1.473, *p* = 0.2147).

The lack of strong activity by the *E. macmahonii* venom in toad plasma suggests that, overall, the venom is not specialised for acting on toad physiology, at least in the case of coagulant activity. Despite limited information on the feeding ecology of *E. macmahonii* and a small number of amphibian species being sympatric with this viper, niche partitioning may make it unlikely that toads form an important dietary item, as amphibians are rare in the shifting, sandy dunes it occupies. *Eristicophis macmahonii* is said to occasionally consume rodents [2,8,18,34], and while rodent plasma was not tested here, rodents and humans share considerable similarity in plasma proteins as well as blood factor sequence identity and clotting times [35,36,37]. It is therefore possible that procoagulant venom activity functions to aid the capture of rodent prey. However, it is interesting to note that this venom also had a rapid procoagulant effect on chicken plasma, greater even than its effect on human plasma, despite birds thought to only be occasional prey items [2,8,18,34]. This may be due to a conserved mechanism of action. Despite conjectures that a diet dominated by endothermic prey may select for haemotoxic venom [38,39], these results are contrary to this hypothesis as the venom of *E. macmahonii*—a generalist that includes large proportions of ectotherms such as lizards and arthropods—is predominantly haemotoxic. In contrast, that of *P. fieldi*—a generalist that appears to prefer birds and rodents—is dominated by neurotoxins. Such “ectothermic/endothermic prey” inferences are likely a major over-simplification and would benefit from greater incorporation of individual species’ ecologies.

*Pseudocerastes urarachnoides* venom possessed rapid, taxonomically “broad-spectrum” procoagulant activity, significantly increasing both clot initiation speed and clot formation rate in each plasma tested (Table 1). There were moderate reductions of clot strength in the human and chicken plasmas, however, that were not observed in the thrombin- and factor X-induced clots. This suggests that the venom may be concurrently cleaving fibrinogen in a degradative manner. It is noteworthy that the venom had strong procoagulant activity on the toad plasma. This result was unexpected as toad plasma demonstrated generally low reactivity and *P. urarachnoides* is said to feed exclusively on birds. However, as the cleavage site of factor X is highly conserved, even between such divergent lineages as amphibians and placental mammals [40], this broad coagulant activity may be due to activation of factor X homologues via their conserved molecular sites. Alternatively, as the diet of juvenile *P. urarachnoides* is largely unknown, toads may form an important part of their diet. Future studies assessing the venom composition of juvenile snakes in parallel with their ecology are required.

In human plasma thromboelastograms, *P. fieldi* and *P. persicus* venoms prevented a clot from forming. Thromboelastography provides a more biologically accurate measure of clotting function than the previously described clotting assays. These results therefore confirm that the slow clotting times and weak zymogen activation observed in the previous assays for these species are associated with weak, friable clots. In the chicken plasma, clot initiation period was lengthened by around 20% by both venoms; however, the ensuing clots were comparable to (*P. persicus*) or greater than (*P. fieldi*) the spontaneous clotting control in both formation rate and strength. Thus, although clot formation was slightly delayed, clotting function of chicken plasma was fundamentally uncompromised by these two venoms. Given that these species are known to consume a high proportion of avian prey [2,8,19], this suggests that venom-plasma interactions may contribute little to avian subjugation. However, the reduction in clot initiation period may facilitate the dispersal of neurotoxic PLA_2_s that dominate *P. fieldi’s* venom. Notably, the venoms completely inhibited clotting of toad plasma (Figure 8), despite that anticoagulant venom activity was not evident in chicken plasma. Anticoagulant venom activity is common amongst amphibian specialists [38,41], suggesting that it is a successful means of subjugating toads, which are otherwise renowned for their resistance to many toxin groups [42]. Where the toxins inducing this action have been identified in such studies, they are typically PLA_2_s [38], and both *P. fieldi* and *P. persicus* venom have these toxins in considerable abundance. Though amphibians are not reported as prey for adult *P. fieldi* or *P. persicus*, ecological studies are lacking. While some studies suggest that *P. fieldi* and *P. persicus* adults prefer feeding on birds, these conclusions are drawn on account of feathers being common in faeces. However, feathers are not easily digestible in contrast to soft-bodied toads and this may be biasing such conclusions. It is therefore hypothesised that this observed anticoagulant activity in toad plasma reflects an ecological adaptation for amphibian predation. However, an in-depth analysis of the vipers’ venoms along with their ecology is required to investigate this hypothesis adequately.

Crucially, these thromboelastograms raise concerns regarding the appropriateness of building evolutionary models and making inferences on snake ecology and predator-prey coevolution upon data obtained using only human plasma and crude venoms. This is particularly true for analyses focussed on species that adopt dietary generalism or prey upon non-mammalian species. The extreme variation between plasmas observed here suggest that evolutionary inferences made in such studies may ultimately prove misguided. Care should be taken to contain such conclusions within the bounds of the chosen model.

## 3. Conclusions

The most striking observations from this study are i) the substantial divergences in the bioactivities of these species’ venoms, despite that all members of the clade superficially share many similarities in their ecology; and ii) the lack of phylogenetic patterns associated with these activities, despite that there is typically similarity between the venom profiles of closely related snakes.

Overall, this clade of closely related vipers presents an unusual example of venom variation. The dietary generalism of *P. fieldi* is paired with a seemingly specialised proteome and venom activity (i.e., neurotoxic-dominant), while the dietary generalism of *P. persicus* and *E. macmahonii* is paired with diverse venom biochemistry. In contrast, the diverse venom proteome of *P. urarachnoides* is paired with a specialised diet and venom activity (i.e., coagulating-dominant). These findings superficially deviate from suppositions that indicate dietary generalism favours toxinological diversity [43] and evade any overarching evolutionary trends. For example, *P. persicus* venom shares greater proteomic and functional similarity to *P. fieldi* than to *P. urarachnoides* with whom it is sister. Furthermore, the venom proteomes of *P. urarachnoides* and *E. macmahonii*, which diverged around 16–18 Mya, most closely resemble that of typical vipers and thus represent the posited plesiotypic condition of the clade [21]; yet, functionally speaking, both *P. urarachnoides* and *P. fieldi* venoms present an apotypic state—*P. fieldi* more so if both venom function and proteome are considered. However, *P. urarachnoides* arguably presents the most derived predatory characters, should venom function be considered in parallel with morphological traits.

Given the conjectured shared (though highly variable) expression of neurotoxic PLA_2_s and procoagulant toxins within the clade, the common ancestor of this group may have possessed a venom most resembling that of *E. macmahonii*. Unfortunately, the lack of consistent patterns between venom proteome and bioactivity in relation to these species’ phylogenetic relationships or feeding ecologies renders any trends of venom evolution obscure, begging the question as to what may be influencing their divergence. Venom variation is commonly linked with populational or age-driven differences in prey availability or endothermic/ectothermic prey groupings [38,39,44]. However, these paradigms appear incongruent with this level of venom variation relative to the overlap in range, age, diet, and/or habitat between these snakes. Other studies investigating venom variation have found no strong link with diet but correlated venom composition with climate [33,45]. The influence of climate may be particularly salient in arid regions, such as the Middle East. The blood chemistry of reptiles is known to fluctuate wildly, depending on metabolic state, temperature, and fasting period [46]. These fluctuations are amplified in desert species, whereby dehydration affects blood volume, osmotic concentration, and pH. The stability of a prey species’ biochemical environment may, in turn, affect venom function and play a key role in driving venom evolution, leading to divergences such as those observed in this study. Such interpretations are unfortunately confounded by a lack of natural history information pertaining to these species. Future studies addressing this evolutionary enigma would benefit from the inclusion of robust dietary data and the integration of broader ecological variables such as morphology along with predation strategies, abiotic factors, and biogeographical history.

Regardless of evolutionary drivers, this study reinforces the notion that phylogenetic relatedness of snakes cannot readily predict venom protein composition or activity, even where overlaps in ecology are evident. This considerable venom variation raises serious concerns regarding the paraspecificity of the Razi polyvalent antivenom, which is specific for *P. persicus*—the venom of which possesses the weakest activity of these species (according to human-oriented in vitro models). Future assessment of antivenom cross-reactivity is therefore crucial.

## 4. Materials and Methods

### 4.1. Venom Samples

All venom work was undertaken under the University of Queensland IBSC approval #IBC134BSBS2015. *Eristicophis macmahonii* venom was collected from 3 adult male snakes from the Nushki district (30.12° N 67.01° E) of Balochistan, Pakistan by SAA. *Pseudocerastes fieldi* and *P. persicus* venoms were purchased from Latoxan. *Pseudocerastes urarachnoides* were collected under the approval of NIMAD # 942485 by PG and BF. Venoms were immediately flash frozen in liquid nitrogen and were subsequently lyophilised and stored at −80 °C until use. Samples were pooled to account for any potential variation in toxin expression between individuals. The lyophilised venoms were reconstituted to a working stock concentration of 1 mg/mL in 50% deionised H_2_O (Milli-Q) and 50% glycerol (Sigma-Aldrich) to prevent freezing and thus reduce enzyme degradation associated with freeze-thaw cycles. Working stock aliquots were stored at −20 °C and used for all subsequent analyses.

### 4.2. Plasma

Human plasma was sourced from the Australian Red Cross (44 Musk Street, Kelvin Grove, Queensland, Australia, 4059) under human ethics approval number 2016000256. Two batches of pooled human plasma (Lot#3379236: 120 mL of Rhesus O+; Lot#3376558: 874 mL of Rhesus AB+) were further combined and aliquoted. The aliquots were flash frozen in liquid nitrogen and stored at −80 °C. Immediately prior to use for experimentation, aliquots were defrosted by immersing in a water bath for 5 min at 37 °C. Clotting time parameters were established for the plasma by measuring clot formation (in triplicate) in the presence and absence of an activator: 46–48 s (s) and 300–500 s, respectively (see “coagulation analyses” for protocol). To check for plasma degradation and ensure the clotting parameters of the plasma aliquots were consistent over time, positive and negative controls were conducted prior to each experimental assay; namely, clotting time was measured in the presence and absence of an activator and compared to pre-established parameters. Chicken and toad bloods were extracted from anaesthetised animals by a trained veterinarian under the University of Queensland Animal Ethics approval SBS/019/14/ARC. Blood was citrated (3.2%) and spun at 15,000 relative centrifugal force (RCF) for 15 min, supernatant (plasma) extracted and aliquoted. The aliquots were flash frozen in liquid nitrogen and stored at −80 °C until use. Immediately prior to use for experimentation, aliquots were defrosted by immersing in a water bath for 5 min at 37 °C.

### 4.3. Proteomics

#### 4.3.1. Venom SDS-PAGE Gel Electrophoresis

One mm 12% SDS-PAGE gels were prepared using the following recipe for resolving gel layer: 3.3 mL deionised H2O, 2.5 mL 1.5 M Tris-HCl buffer pH 8.8 (Tris—Sigma-Aldrich, St. Louis, MO, USA; HCl—Univar, Wilnecote, UK), 100 µL 10% SDS (Sigma-Aldrich, St. Louis, MO, USA), 4 mL 30% acrylamide mix (Bio-Rad, Hercules, CA, USA), 100 µL 10% APS (Bio-Rad, Hercules, CA, USA), 4 µL TEMED (Bio-Rad, Hercules, CA, USA); and stacking gel layer: 1.4 mL deionised H2O, 250 µL 0.5 M Tris-HCl buffer pH 6.8, 20 μL 10% SDS (Sigma-Aldrich, St. Louis, MO, USA), 330 mL 30% acrylamide mix (Bio-Rad, Hercules, CA, USA), 20 μL 10% APS (Bio-Rad, Hercules, CA, USA), 2 µL TEMED (Bio-Rad, Hercules, CA, USA). 10× gel running buffer was prepared using the following recipe: 250 mM Tris (Sigma-Aldrich, St. Louis, MO, USA), 1.92 M glycine (MP Biomedicals, Santa Ana, CA, USA), 1% SDS (Sigma-Aldrich, St. Louis, MO, USA), pH 8.3. For the 1D gels, venom samples were prepared with and without 5% 2-mercaptoethanol (reduction and no reduction of disulphide bridges, respectively), and were boiled at 100 °C for 5 min to denature proteins. Twenty µg venom samples were loaded into the gels and were run in 1× gel running buffer at room temperature for 20 min at 90 V (Mini Protean3 power-pack from Bio-Rad, Hercules, CA, USA) and then 120 V until the dye front approached the bottom of the gel. Gels were then stained with colloidal coomassie brilliant blue G250 (34% methanol (VWR Chemicals, Tingalpa, QLD, Australia), 3% orthophosphoric acid (Merck, Darmstadt, Germany), 170 g/L ammonium sulphate (Bio-Rad, Hercules, CA, USA), 1 g/L coomassie blue G250 (Bio-Rad, Hercules, CA, USA), and de-stained in deionised H_2_O (Milli-Q).

For 2D gels, 300 µg venom samples were loaded onto immobilised pH gradient (IPG) strips (Bio-Rad ReadyStrip, non-linear pH 3–10, 7 cm) and incubated overnight at room temperature. Isoelectric focussing was conducted using a PROTEAN i12 IEF CELL (Bio-Rad Lab) under the following conditions: 50 µA current; 100 V for 1 h, 500 V for 1 h, 1000 V for 1 h, and 8000 V until 98,400 V/h. To reduce and alkylate the samples, IPG strips were incubated in a reducing equilibration buffer (50 mM Tris–HCl, pH 8.8, 6 M urea, 2% SDS, 30% glycerol, 2% DTT) for 10 min, followed by further incubated for 20 min in an alkylating equilibration buffer (50 mM Tris–HCl, pH 8.8, 6 M urea, 2% SDS, 30% glycerol, 2.5% iodoacetamide). The strips were then rinsed with SDS-PAGE running buffer and positioned on top of 12% polyacrylamide gels using 0.5% agarose. Gels were run at 4 °C with a current of 10 mA/per gel for 20 min followed by 20 mA/per gel for the rest of the run until the bromophenol dye front was within 0.5 cm of the base of the gel. After the run, gels were briefly washed with water and stained with 0.2% colloidal Coomassie brilliant blue G250 overnight.

Visible spots were picked from the gel, washed with 100 µL 50 mM ABC twice and destained with 50 mM ABC/50% acetonitrile (ACN), and then incubated with 100% ACN until opaque. Twenty μL of trypsin solution (20 μg trypsin in 100 μL 1 mM HCl and 900 µL of 40 mM ammonium bicarbonate (ABC)) was diluted in 380 μL 40 mM ABC, then 30 µL was added to gel spots and incubated overnight at 37 °C. Subsequently, spots were removed from solution and peptides eluted by adding 1% trifluoroacetic acid (TFA), incubating for 20 min, transferring solution and then repeating with 5% ACN/1% TFA. Solutions were centrifuged at 16,000 RCF for 10 min and supernatant extracted. Solutions were then ziptipped by wetting tip (Millipore ZTC18S096, Sigma-Aldrich, St. Louis, MO, USA) with 100% ACN, equilibrating with 5% ACN/0.1% TFA, loading sample, washing tip with 5% ACN/0.1% TFA, eluting sample with 60% ACN/0.1% TFA, dehydrating sample and then and resuspending in 0.1% formic acid.

#### 4.3.2. Liquid Chromatography-Mass Spectrometry

Peptides extracted from in-gel tryptic digestion were analysed by liquid chromatography-tandem mass spectrometry (LC-MS/MS) using an Agilent Zorbax stable bond C18 column (2.1 mm × 100 mm, 1.8 µm particle size, 300 Å pore size) on a Shimadzu Nexera UHPLC system (Shimadzu, Kyoto, Japan) coupled to an AB SCIEX 5600 Triple TOF mass spectrometer (AB Sciex, Concord, ON, Canada). The chromatographic elution was achieved at a flow rate of 400 µL/min via a gradient of 1 to 40% solvent (90% acetonitrile, 0.1% formic acid) over 4 min. The MS/MS spectra were acquired at a rate of 20 scans per second with a cycle time of 2.3 s, and the resultant 20 most intense ions were obtained and searched against UniProt databanks using Protein Pilot v4.0 software (AB SCIEX, Foster City, CA).

### 4.4. Cytotoxicity

Pancreatic tumour (PaTu-T) cells, melanoma patient derived cells (MM96L), and human neonatal foreskin fibroblasts (NFF) were selected as representatives of both healthy and unhealthy cells from different organs to account for any variation in cell types. Cells were maintained in RPMI medium supplemented with 1% penicillin-streptomycin and 10% foetal bovine serum (FBS). Cells were trypsinised (0.25% trypsin) and seeded 24 h prior to the experiments in 96-well flat bottom plate at a density of 5000 and 3000 cells/well for NFF and MM96L cells, respectively, and at 80% confluence for PaTu-T cells. Plates were incubated at 37 °C in a 5% CO_2_-95% humidified environment. A minimum of three replicates were conducted per treatment. Cell death was visualised via light microscopy using Zeiss Axiovert 200 with a Plan- Apochromat 63 × 1.4 numerical aperture after 24h of treatment with *Pseudocerastes* or *Eristicophis* venoms at 10 µg/mL.

### 4.5. Neurotoxicity

Male chicks (*Gallus gallus*) were euthanized with CO_2_ between 4–10 days after hatching (animal ethics approval number 22575, MARP2 committee, Monash University). Replicates were run using tissues of different ages to account for differences in effects related to tissue development. Chick biventer cervicis nerve-muscle were dissected and preparations were mounted under 1 g tension in 5 mL organ baths containing physiological salt solution (in mM; 118.4 NaCl, 4.7 KCl, 1.2 MgSO_4_, 1.2 KH_2_PO_4_, 2.5 CaCl_2_, 25 NaHCO_3_, and 11.1 glucose) at 34 °C and bubbled with carbogen (95% O_2_; 5% CO_2_). Electrodes were placed around the tendon of the biventer muscle. Electrical stimulation of the motor nerve (0.2 ms duration, 0.1 Hz, supramaximal V) using a Grass S88 stimulator (Grass Instruments, Quincy, MA, USA) evoked indirect twitches. Selective stimulation of the nerve was confirmed by the abolition of twitches with d-tubocurarine (10 µM), a nicotinic acetylcholine receptor (nAChR) competitive antagonist. Tissues were then washed repeatedly with physiological salt solution to restore twitch responses to nerve stimulation. The stimulation was ceased, and the contractile responses to acetylcholine (1 mM ACh for 30 s), carbachol (20 µM CCh for 60 s), and potassium chloride (KCl; 40 mM for 30 s) were obtained and recorded. The organ bath was then washed, and electrical stimulation was resumed and maintained for 30 min to allow the preparation to equilibrate. Venom (10 µg/mL) was added to the organ bath and the twitch height was recorded until twitch response was abolished. If twitch response did not cease, measurements were stopped at 60 min. The stimulator was turned off again and the bath was washed. Contractile responses to ACh, CCh, and KCl were obtained again to compare with responses prior to venom addition. The twitch responses to electrical stimulation and contractile responses to agonists (ACh, CCh, and KCI) were measured using a Grass FT03 force displacement transducer (Grass Instruments, Quincy, MA, USA) and recorded on a PowerLab system (ADInstruments Pty Ltd., Bella Vista, NSW, Australia). At least four replicates were run for each venom.

### 4.6. Coagulation Analyses

#### 4.6.1. Clot Formation Time

Coagulant activity was assessed by incubating the venoms with human plasma and measuring time until clot formation. The coagulation analyses were performed using a Stago STA-R Max^®^ automated coagulation analyser and Stago Analyser software v0.00.04 (Stago, Asnières sur Seine, France). Venom working stock was manually diluted to 0.1 mg/mL in Owren Koller (OK) Buffer (Stago Cat# 00360) and loaded into the analyser. All subsequent dilutions, steps, and measurements were automated. Venom-induced clotting times were measured over a 9-point dilution series (µg/mL: 30.00, 20.00, 10.00, 4.00, 1.67, 0.67, 0.25, 0.13, and 0.05). Assay conditions were as follows: 50 µL venom (further diluted in OK Buffer, as appropriate, to achieve desired concentration) was incubated with 50 μL of CaCl_2_ (0.025 M, Stago Cat# 00367), 50 µL phospholipid (cephalin prepared from rabbit cerebral tissue from STA C.K. Prest standard kit, Stago Cat# 00597, solubilised in 5 mL OK Buffer), and 25 μL of OK Buffer for 120 s at 37 °C. 75 µL of human plasma was then added and time until clot formation measured by the analyser using a viscosity-based detection system. Each dilution series was repeated in triplicate. To measure toxin co-factor dependency, the above assay was repeated in the presence and absence of calcium and/or phospholipid. Conditions were identical except for 50 µL OK buffer being used in place of the absent co-factor in order to maintain a consistent final volume (250 μL). Plasma negative controls were conducted under identical conditions except for 50 μL of blank solution (v/v: 5% glycerol, 5% H_2_O, 90% OK buffer) substituting the venom. Positive controls were conducted by incubating 50 μL Kaolin (STA C.K. Prest standard kit, Stago Cat#00597) with 50 µL plasma for 120 s at 37 °C. 50 μL CaCl_2_ (0.025 M, Stago Cat#00367) was then added and time until clot formation measured.

#### 4.6.2. Factor X and Prothrombin Activation

To measure the activation of the coagulation factor Factor X (FX) by the venom, 10 µL venom (containing 0.05 µg, 0.1 μg, and 0.5 µg dry venom weights) was plated in at least triplicate on a 384 well plate along with 10 µL FX (Haematologic Technologies, Inc., 57 River Road, Unit 1021, Essex Junction, VT 05452 USA) (0.1 µg dry weight) and 10 µL phospholipid (cephalin prepared from rabbit cerebral tissue from STA C.K. Prest standard kit, Stago Cat#00597, solubilised in Owren Koller (O.K.) Buffer). To account for direct substrate cleavage by the venom, 10 µL venom (containing 0.05 µg, 0.1 µg, and 0.5 µg dry venom weights) was plated in triplicate on a 384-well plate along with 10 µL phospholipid and 10 µL buffer (150 mM NaCl, 50 mM Tris-HCl, pH 7.4) to substitute the FX for later subtraction. Ten µL (0.1 µg dry weight) of FXa (Haematologic Technologies, Inc via United Bioresearch, Sydney.) in at least triplicate was used as a positive control. Negative control consisted of 20 µL dilution buffer and 10 µL phospholipid. Activity was measured by adding 70 µL quenched Fluorogenic Peptide Substrate Cat#ES002 (R and D systems, Minneapolis, Minnesota, USA) (10 µL substrate in 5mL enzyme buffer: 150 mM NaCl, 50 mM Tris-HCl, 5 mM CaCl, pH 7.4). Fluorescence was monitored (excitation at 390 nm and emission at 460 nm) at 37 °C over 400 min or until activity ceased. To measure prothrombin activation, test conditions were identical except prothrombin (Haematologic Technologies, Inc.) was used in place of FX, thrombin (Haematologic Technologies, Inc.) in place of FXa, and Fluorogenic Peptide Substrate Cat#ES011 (measured at 390 nm/460 nm) in place of substrate Cat#ES002. Data were normalised against no reaction (0%) and the positive control (100%) using GraphPad Prism version 8.0.0 for iOS (GraphPad Software, San Diego, CA, USA, www.graphpad.com).

#### 4.6.3. Thromboelastography

The effects of venoms on clot dynamics of plasma were measured using a Thromboelastograph^®^ 5000 Haemostasis analyser (Haemonetics^®^, Haemonetics Australia Pty Ltd., Sydney, Australia). This machinery consists of a stationary pin suspended by a torsion wire into the cup. The cup oscillates, and as the clot develops the changes in the viscoelasticity of the fluid are detected by the pin and wire, which act as a torque transducer and generates a “trace” output. For the experiments, 189 µL of human, chicken, or toad plasma was combined with 72 μL CaCl_2_ (25mM stock solution Stago Cat# 00367 STA), 72 μL phospholipid (STA C·K Prest standard kit, Stago Cat# 00597; solubilised in Owren Koller (OK) buffer), and 20 μL OK buffer in an assay cup (Haemonetics Australia Pty Ltd., North Rye, Sydney, Australia). In the final step, 7 µL venom (1 mg/mL in 1:1 glycerol:deionised H_2_O) was added, pipette mixed, and then the machine was immediately started. Tests were run at 37 °C and measured for 30 min. Negative and positive controls followed identical methods, except 7 µL of 1:1 glycerol:deionised H_2_O (negative control) and 7 µL thrombin, FXa (STA^®^-Liquid Anti-Xa, Stago), and/or kaolin (STA C.K. Prest standard kit, Stago Cat# 00597) (positive controls) replaced the 7 µL venom in the final step. As these plasmas had low levels of platelets, values attained in these assays are not comparative to in vivo coagulation. For example, clot strength is compromised by the lack of fibrin-binding platelets, which makes assessment of the venoms’ influence on such parameters difficult. However, blood factors that are upstream in the coagulation cascade, such as FXII, are known to spontaneously convert into their active form. This initiates the coagulation cascade despite the absence of an activator, and the resulting “spontaneous” clot is structured according to the native proportions of blood factors in the platelet-poor plasma used in in vitro assays. The venom data were therefore normalised against the spontaneous clotting control data (spontaneous clot: 0%; potential maxima: 100%) to assess the venoms’ influence on in vitro coagulation cascade kinetics.

### 4.7. Statistics

Data were graphed and analysed using GraphPad Prism version 8.0.0 for iOS (GraphPad Software, San Diego, California, USA, www.graphpad.com). Venom neurotoxicity data were normalised against the controls and tested for normality using Shapiro-Wilk, Kolmogorov Smirnov, and Q-Q plots. Contractile heights at 60 min were compared via one-way ANOVA with Tukey’s *post hoc* and paired t-tests (control vs. venom) using Prism default settings with 95% confidence interval. To analyse the data obtained in the Stago coagulation analyses, dose-response plasma coagulation curves were normalised against maximal response of the venom’s clotting time (100%) and blank plasma control spontaneous clotting time (0%). EC_50_s were then calculated using the non-linear regression (log)dose vs. (normalised)response model—variable slope with Shapiro–Wilk, Kolmogorov Smirnov, and Q-Q plot diagnostic tests for normality and confidence intervals set to 95%. Clotting times were compared using one-way ANOVA (grouped analyses) with Tukey’s post hoc analysis or unpaired T-tests using Prism default settings with a 99% confidence interval. Thromboelastography data were tested for normality using Shapiro-Wilk, Kolmogorov Smirnov, and Q-Q plot diagnostic tests. The data were normalised against the spontaneous clotting control data for each plasma (spontaneous clot time: 0%; potential maxima: 100%). As positive controls (thrombin and FXa) were of human origin, they could not be used to obtain maximal values of clotting parameters against which to normalise venom-induced clot data. “Potential maxima” values for clot maximum amplitude and angle for chicken and toad plasma were therefore obtained and estimated from previous studies that characterised the clotting parameters of alike animal plasmas under comparable methodologies [47,48,49]. Venom-induced values were compared to spontaneous controls using unpaired t-tests with 99% confidence intervals.

## Figures and Tables

**Table 1:** Differences between means (±SD) of human, toad, and chicken plasma controls versus those with *P. urarachnoides* venom measured via thromboelastography.

*P. urarachnoides* Venom vs. Spontaneous Control Difference Between Means ± SD (%); Unpaired t-Test		
Plasma (*n* = 4)	Clot Initiation Speed	Clot Formation Rate
Human	95 ± 4.3%; t (6) = 22.08, *p* < 0.000001	61 ± 14.8%; t (6) = 4.08, *p* = 0.00651
Toad	99 ± 4.3%; t (6) = 23.15, *p* < 0.000001	63 ± 2.6%; t (6) = 24.37, *p* < 0.000001
Chicken	96 ± 4.3%; t (6) = 22.46, *p* < 0.000001	38 ± 10.9%; t (6) = 3.519, *p* = 0.01254

## Data Availability

Raw data is included in the Supplementary Datasheets.

## Supplementary Materials

The following are available online at https://www.mdpi.com/2072-6651/13/2/112/s1, Table S1: Toad plasma TEG, Table S2: D. russelii clot time, Sheet S1: P. ura MS data.
